# Molecular profile of the NF‐κB signalling pathway in human colorectal cancer

**DOI:** 10.1111/jcmm.17545

**Published:** 2022-11-25

**Authors:** Maria Dobre, Bogdan Trandafir, Elena Milanesi, Alessandro Salvi, Ioana Alina Bucuroiu, Catalin Vasilescu, Andrei Marian Niculae, Vlad Herlea, Mihail Eugen Hinescu, Gabriel Constantinescu

**Affiliations:** ^1^ Victor Babes National Institute of Pathology Bucharest Romania; ^2^ Faculty of Medicine Carol Davila University of Medicine and Pharmacy Bucharest Romania; ^3^ Fundeni Clinical Institute Bucharest Romania; ^4^ Division of Biology and Genetics, Department of Molecular and Translational Medicine University of Brescia Brescia Italy; ^5^ Clinical Emergency Hospital Bucharest Bucharest Romania

**Keywords:** colorectal cancer, gene expression, miRNAs, NF‐κB

## Abstract

The development and progression of colorectal cancer (CRC) have been associated with inflammation processes that involve the overactivation of the NF‐κB signalling pathway. The characterization of the NF‐κB expression profile in CRC is an important topic since the suppression of NF‐κB represents a potential therapeutic approach. In this study, we assessed the expression levels of 84 NF‐κB‐related genes in paired tumoral (T) and peritumoral (PT) tissues from 18 CRC patients and 18 normal colonic mucosae, and the expression levels of three miRNAs targeting the most dysregulated genes revealed by the case–control analysis. Comparing the gene expression profile of T and controls, 60 genes were dysregulated. The comparison of T and PT revealed 17 dysregulated genes in the tumoral tissues, with *IL1B*, *CXCL8*, *IL1A*, and *CSF2* being the most upregulated. Notably, through a bioinformatics analysis, the differential gene expression of 11 out of the 17 genes was validated on a larger cohort of 308 CRC patients compared with 41 controls. Moreover, a decrease in the levels of *RELA*, *NOD1*, *CASP8*, *BCL2L1*, *ELK1*, and *IKBKB* was identified in poorly differentiated tumours compared to moderately differentiated tumours. The analysis of the three miRNAs targeting *IL1B*, *CXCL8*, *IL1A*, and *CSF2* showed that miR‐182‐5p was upregulated in T compared with PT, whereas miR‐10b‐5p was downregulated in T compared with PT and control tissues. Our results may contribute to the design of new experimental therapeutic strategies based on endogenous molecules, such as miRNAs, to target the genetic key players of the NF‐ κB pathway.

## 1 BACKGROUND

Colorectal cancer (CRC) represents the third most diagnosed malignancy and the second cause of cancer‐related deaths in the world, with 1.9 million new cases and nearly 900,000 related deaths in 2020.^1^ The traditional classification of colorectal malignancies is based on anatomical and histological factors, including localization, histological differentiation, locoregional invasiveness, and distant metastasis.^2^ However, this traditional paradigm has shifted toward transcriptome‐based classifications for the identification of molecular subtypes, also integrating genomic and proteomic data obtained by basic research, bioinformatics, and the clinical setting.^3^ The presence of specific mutations, aberrant DNA methylation in the promoters of specific genes, and microsatellite instability constitute key elements necessary for the stratification of patients and the choice of the therapeutic regimen.^4^, ^5^, ^6^, ^7^

In the last two decades, the development and progression of different types of cancer have often been associated with the immune system and inflammation processes.^8^ Inflammation, cell growth, survival, and cellular development are regulated by the nuclear factor kappa B (NF‐ κB), which controls the immune response at various stages by modulating the expression of important regulatory genes acting as a transcription factor complex.^9^ NF‐κB family comprises five transcription factors: RelA/p65, RelB, c‐Rel, NF‐κB1(p50/p105), and NF‐ κB2(p52/p100). These act as dimers in two distinct but interdependent arms of the NF‐κB pathway: the canonical and the non‐canonical pathways.^10^

The canonical NF‐κB signalling pathway is activated by ligands of cytokine receptors, such as IL1R1, some TNF receptor (TNFR) superfamily members, T‐cell receptors, or B‐cell receptors, which phosphorylate the IKK complex, causing the translocation of p65 and p50 heterodimers into the nucleus where they activate gene expression. In contrast, the non‐canonical NF‐κB pathway selectively responds to a specific group of stimuli that includes ligands of a subset of TNFR superfamily members such as LTβR, BAFFR, CD40, and RANK.^11^ The phosphorylation of the IKK complex leads to the phosphorylation of p100, inducing its proteasomal processing to p52. After the transformation of p100 to p52, the p52/RelB dimer translocates to the nucleus and promotes gene transcription.

NF‐κB is known to have an important role in several cancers including liver, pancreatic, prostate cancers,^12^ small‐cell‐lung cancer, and renal carcinoma, where it is associated with poor prognosis, and ovarian cancer where it promotes chemoresistance, cancer stem cell maintenance, metastasis, and immune evasion.^13^ The activation of the canonical pathway has been well established for antiapoptotic and immunomodulatory functions in response to the tumour microenvironment, whereas the non‐canonical pathway is involved in cancer stem cell maintenance and tumour reinitiation.^13^

A growing body of evidence supports the role of NF‐κB in gastric and colorectal cancers, which classically depend on inflammation. Chronic gastrointestinal inflammation occurring in inflammatory bowel diseases and chronic inflammation caused by *Helicobacter pylori* and primary sclerosing cholangitis have been long associated with an increased risk of CRC.^14^

In sporadic CRC, elevated expression of the pro‐inflammatory cytokines IL‐8, IL‐23a, IL‐1a, IL‐1b, IL‐17a, INFγ, and IL‐6 has been identified compared to adjacent non‐cancerous tissues.^15^ In line with this, IL‐8 mRNA and protein expression was significantly upregulated in pathological colorectal entities compared with neighbouring tissues and was associated with induction and progression of CRC.^16^ Moreover, IL‐23 may indirectly promote tumour cell survival, since it drives intestinal inflammation by inducing other pro‐inflammatory cytokines, such as IL‐6, IL‐17, and IL‐22.^17^ Due to the aberrant NF‐κB activity in CRC, pharmacological inhibitors of NF‐κB against cancer initiation or progression are being developed as a novel therapeutic approach for better management of CRC.^18^

MicroRNAs (miRNAs) play key roles in CRC progression, having a dual role in both oncogenesis and tumour suppression. Also, the CRC mutational status is associated with a specific miRNA profile.^19^ Moreover, miRNAs modulate the NF‐κB signalling pathway in cancer acting on the mRNA level, regulating the expression of proteins at different levels of the pathway. Several miRNAs contain experimentally verified NF‐κB binding sites in their promoters that have been validated as targets of NF‐κB.^20^ These miRNAs, implicated in the modulation of innate immune responses and inflammation, display either pro‐ or anti‐ inflammatory functions.^21^

The aim of the present study was (i) to evaluate the NF‐κB signalling pathway gene expression in paired tumoral and peritumoral tissues from patients with CRC compared with normal colonic tissues by analysing the expression levels of 84 key transcripts, and (ii) to evaluate the expression levels of miRNAs targeting the most dysregulated genes as revealed by the case–control analysis.

## 2 METHODS

### 2.1 Patients

Eighteen tumoral (T) and the corresponding peritumoral (PT) surgical tissues were collected from patients with primary CRC during tumour surgical resection performed by a single team at the “Fundeni” Clinical Institute in Bucharest, Romania. The peritumoral tissue was collected approximately 8 to 10 cm away from the tumours. Eighteen biopsies from control patients presenting not affected colonic mucosa (CTRL) were collected during colonoscopy screenings. The exclusion criteria for controls were the following: (1) presence of CRC and/or inflammatory bowel diseases, (2) treatments with nonsteroidal anti‐inflammatory drugs within the past 3 months, and (3) treatment with anticoagulants or antiplatelets within the past 3 months. For RNA preservation, the collected tissues have been immersed in RNA protect Tissue Reagent (Qiagen, Hilden, Germany) for 48 to 72 h. After the removal of this solution, the tissues were stored at −80°C until RNA isolation. All samples were examined by an experienced pathologist who defined the grading (G) and the TNM (T = size of the primary tumour; N = degree of spread to regional lymph nodes; M = presence of distant metastasis) stage. The present study was approved by the Ethics Committee of the “Victor Babes” National Institute of Pathology (approval number 78, December 3, 2019) and the Ethics Committee of the “Fundeni” Clinical Institute (December 11, 2019) and was conducted in accordance with the Code of Ethics of the World Medical Association (Declaration of Helsinki). For all patients, the signed informed consent was obtained before sample collection, and the following biochemical data were registered: haemoglobin (g/dl); white blood cells‐WBC‐ (*N*/μl); platelets (*N* × 10^3^/μl); international normalized ration –INR‐; fibrinogen (mg/dl); albumin (g/dl). Moreover, three tumoral markers were evaluated including carcinoembryonic antigen –CEA‐(ng/ml), carbohydrate antigen ‐CA19‐9 (U/ml), and alpha‐fetoprotein –AFP‐ (ng/ml). Moreover, the presence of following comorbidities has been recorded: liver cirrhosis, cardiovascular disorders, obesity, and diabetes.

### 2.2 Gene expression analysis

Total RNA was isolated using the miRNeasy Mini Kit (Qiagen, Hilden, Germany), and 600 ng of RNA were reverse‐transcribed with the RT^2^ First Strand Kit (Qiagen, GmbH) according to the manufacturer's protocols. The expression of 84 inflammatory genes was evaluated with the RT^2^ Profiler™ PCR Array Human NF‐κB Signalling Pathway (PAHS‐025Z, Qiagen, GmbH) and RT^2^ SYBR Green ROX qPCR Mastermix (Qiagen, GmbH) using an ABI‐7500 fast instrument (Thermo Scientific, USA). This panel includes 84 laboratory‐verified qPCR assays involved in the NF‐κB pathway at different levels and comprises: ligands and receptors, cytoplasmic sequestering/releasing of NF‐κB, transcription factors, genes implicated in downstream signalling, general immune response and apoptosis, as well as other factors involved in the NF‐κB pathway (Table S1). The expression levels of each gene were normalized against the geometric mean of *RPLP0* and *HPRT1*. The 2^−ΔCT^ mean values and the fold change (FC = 2^−ΔΔCT^) were calculated as previously described.^22^ The FC values (2^−ΔΔCt^) were calculated using as reference different groups according to the comparison: FC (T vs PT) = 2^−ΔCt^ T/2^−ΔCt^ PT; FC (T vs CTRL) = 2^−ΔCt^ T/2^−ΔCt^ CTRL; FC (PT vs CTRL) = 2^−ΔCt^ PT/2^−ΔCt^ CTRL; FC (G3 vs G2) = 2^−ΔCt^ G3/2^−ΔCt^ G2. The two housekeeping genes used for normalization were selected using the RefFinder algorithm (http://leonxie.esy.es/RefFinder/)^23^ among five candidates genes that are included in the array (*ACTB*, *B2M*, *GAPDH*, *HPRT1*, and *RPLP0*).

### 2.3 Validation of gene expression results in TCGA database

The transcripts found significantly differentially expressed in our study comparing 18 T vs 18 PT tissues have been investigated in a larger cohort using public databases. To this aim, the individual gene expression data obtained by RNA‐seq from 308 patients with colon adenocarcinoma (from TCGA database) and 41 controls (from GTEx study) have been downloaded and analysed using OncoDB tool (http://oncodb.org/index.html).^24^

### 2.4 In silico miRNA selection and expression analysis

Based on the gene expression results of this study, in which we identified *IL1B* and *CXCL8* as the most upregulated genes in the T vs PT comparison, and *EGR1* and *CCL2* as the most down regulated, we selected three candidate miRNAs targeting these genes. These miRNAs were identified through DIANA‐TarBase v.8,^25^ and their role in CRC was confirmed by literature data. Based on this survey, the expression of the following miRNAs was analysed: miR‐20a‐5p, miR‐10b‐5p, and miR‐182‐5p. Total RNA (10 ng) was reverse‐transcribed with the miRCURY LNA RT Kit (Qiagen, GmbH). The expression levels were evaluated using the miRCURY LNA SYBR Green PCR Kit and the miRCURY LNA miRNA PCR Assay (Qiagen, GmbH). The Ct data were normalized against the geometric mean of two reference miRNAs (*SNORD38B* and *SNORD49A*).

### 2.5 Statistical analysis

The values of mRNA and miRNA levels were not normally distributed (Shapiro–Wilk test, *p* < 0.05), thus, non‐parametric tests were applied. For the related samples, the Wilcoxon signed‐rank test was applied to assess the differences between paired tumoral and peritumoral tissues, while the Mann‐Whitney test was used to compare controls and patients. Correlations between gene expression and clinical parameter levels were calculated by Pearson test. The differences in mRNA or miRNA levels between the groups were considered significant when *p* < 0.05 and 0.5 ≥ FC ≥ 2. Differences in age and sex between controls and patients were tested with the *t*‐test and the chi‐squared test, respectively. The Statistical Package for the Social Sciences (SPSS version 20.0) and the GraphPad Prism 8.4.3. were used to perform statistical analysis and generate the graphs.

## 3 RESULTS

In the demographic analysis, the two groups of patients and controls were homogeneous for age (*p* = 0.127) and sex distribution (χ^2^ = 0.444; *p* = 0.505). The clinical data of the patients with CRC are summarized in Table 1. Fifteen patients presented comorbidities including cardiovascular diseases (*N* = 12), liver cirrhosis (*N* = 2) and cardiovascular disease, diabetes, and obesity were present in one patient (*N* = 1). In most of the patients, the tumour was localized in the ascending colon (*N* = 6), the sigmoid (*N* = 5), and the rectum (*N* = 5). The grade of tumour ranged from G1 (well‐differentiated) to G3 (poorly differentiated), with 11 patients presenting G2 (moderately differentiated). Nine patients presented regional lymph node involvement, without the presence of distant metastases.

**TABLE 1:** Clinical data of the patients involved in the study

Features	Tumoral (*N* = 18)
Age (mean ± SD)	67.77 ± 8.84
Sex	8F; 10 M
Liver cirrhosis (*N* affected)	2
Cardiovascular disorders (*N* affected)	13
Obesity (*N* affected)	1
Diabetes (*N* affected)	1
Localization	Ascending colon (*N* = 6) Descending colon (*N* = 1) Sigmoid (*N* = 5) RSJ (*N* = 1) Rectum (*N* = 5)
Grading	G1 (*N* = 2) G2 (*N* = 12) G3 (*N* = 4)
TNM	T2 N0 M0 (*N* = 1); T3 N0 M0 (*N* = 7); T3 N1a M0 (*N* = 2); T3 N1b M0 (*N* = 3); T3 N2a M0 (*N* = 1); T3 N2b M0 (*N* = 1); T4a N2b M0 (*N* = 1); T4b N0 M0 (*N* = 1); T4b N1 M0 (*N* = 1);
Haemoglobin (g/dl) mean (min–max)	11.91 (7.1–17.0)
WBC (*N*/μl) mean (min–max)	7570 (4600–14,800)
Platelets (N10^3^/μl) mean (min–max)	314 (137–777)
INR mean (min–max)	1.02 (0.82–1.22)
Fibrinogen (mg/dl) mean (min–max)	458 (275–796)
Albumin (g/dl) mean (min–max)	4.0 (3.1–5.5)
CEA (ng/ml) mean (min–max)^a^	40.8 (0.48–444)
CA 19–9 (U/ml) mean (min–max)^a^	28.3 (0–211)
AFP (ng/ml) mean (min–max)	103.3 (1.38–1000)

Abbreviations: AFP, Alpha‐Fetoprotein; CA 19–9, Carbohydrate Antigen 19–9; CEA, Carcino‐Embryonic Antigen; INR, International Normalized Ratio; WBC, White Blood Cells.

^a^ Data available for 15 patients; TNM (T = size of the primary tumour; N = degree of spread to regional lymph nodes; M = presence of distant metastasis).

**FIGURE 1:**
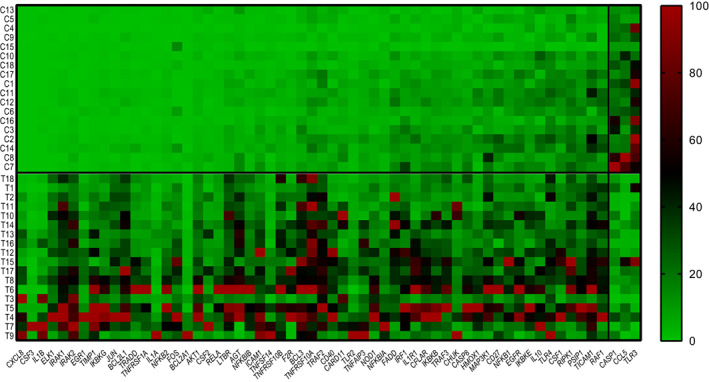
The heat map shows the expression patterns of the 60 genes out of 84 found dysregulated in T vs CTRL tissues with *p*  < 0.05 and 0.5 ≥ FC ≥ 2. Red colour indicates upregulation, green downregulation, and black refers to the not modified gene expression. After normalization, genes were ordered according to the expression levels and were scaled considering the highest value as 100% and the lowest as 0%. The rows refer to individual tissue samples and the columns to the gene symbols

We first compared the expression levels of the 84 candidate genes between the T and CTRL tissues, finding 60 significantly dysregulated genes (57 upregulated and 3 downregulated) within the tumoral tissue (Figure 1).

The dysregulated genes belong to the IL1R and TLR signalling pathways, which activate the canonical NF‐κB pathway, and the TNF, CD40, and LTB signalling pathways, which mediate the activation of the non‐canonical NF‐κB pathway. Key genes of these two pathways were dysregulated and included the following: *NFKB1*, *NFKBIA*, *IKBKB*, *IKBKG*, *RELA* (canonical), *NFKB2*, *IKBKE*, and *TRAF3* (non‐canonical). Among the most upregulated genes in the tumoral tissues were *CXCL8* (induced by the pro‐inflammatory cytokines IL1B), *CSF3*, *IRAK1*, *IRAK2*, and *ELK1*. Notably, *IL‐1B*, *IRAK1*, and I*RAK2* belong to the IL1R signalling pathway that is activated in the CRC tissues. The three downregulated genes were *CASP1*, *CCL5*, and *TLR3*.

No difference was found when we stratified the patients according to the tumour localization. A decrease in the levels of *RELA*, *NOD1*, *CASP8*, *BCL2L1*, *ELK1*, and *IKBKB* was identified in the poorly differentiated (G3) tumours compared with the moderately differentiated (G2) ones (Table 2). *ELK1* was also downregulated in tumoral tissues from the patients with regional lymph nodes involvement compared with those without (FC = 0.50, *n* = 9, *p* = 0.031) (data not shown). Moreover, positive correlations between CA19‐9 and IL‐1B, and CXCL8 gene expression levels (*p* = 0.033, *r* = 0.553; *p* = 0.008, *r* = 0.658, respectively) and between CEA and CXCL8 levels (*p* = 0.017, *r* = 0.605) were observed.

**TABLE 2:** Genes found downregulated between G3 and G2 tumours (0.5 ≥ FC ≥ 2; *p* < 0.05). Genes are listed according to ascending FC.

Genes	G3 vs G2	
	*p*‐value^a^	FC
*RELA*	0.042	0.34
*NOD1*	0.020	0.37
*CASP8*	0.020	0.38
*BCL2L1*	0.004	0.40
*ELK1*	0.030	0.51
*IKBKB*	0.042	0.47

Abbreviations: FC, Fold Change; G2, Tumour grade 2; G3, Tumour grade 3.

^a^ Mann‐Whitney test.

**FIGURE 2:**
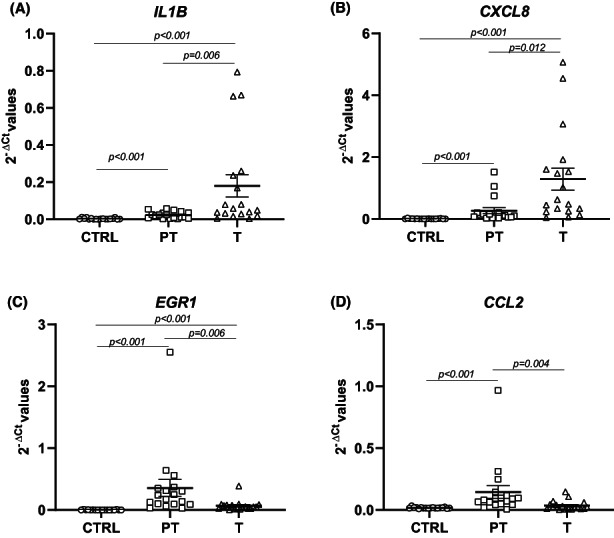
(A) Increased *IL1B* mRNA levels in tumoral tissue vs peritumoral and control; (FC = 7.79 and FC = 55.98, respectively) (B) Increased *CXCL8* mRNA levels in tumoral tissue vs peritumoral and control; (FC = 4.77 and FC = 205.01, respectively). (C) Decreased *EGR1* mRNA levels in tumoral tissue vs peritumoral (FC = 0.19) and increased in tumoral tissue vs control (FC = 24.71). (D) Decreased *CCL2* mRNA levels in tumoral tissue vs peritumoral (FC = 0.24). Data are presented as 2^−∆Ct^ values. Bars represent the expression averages±standard error of mean (SEM). The p‐value was calculated with Wilcoxon signed‐rank test for the comparison T vs PT and with the Mann–Whitney U test for the comparisons T vs CTRL and PT vs CTRL

When we compared the gene expression between the T and PT paired tissues, we identified a list of 17 dysregulated genes (7 upregulated and 10 downregulated). The most upregulated genes were *IL1B*, *CXCL8*, and *IL1A*, and the most downregulated ones were *EGR1*, *CCL2*, and *TLR3* (Table 3 and Figure 2). These 17 genes were also significantly dysregulated when both T and PT were compared with CTRL (Table 3). Notably, the differential gene expression of 11 out of the 17 genes was validated on a larger cohort of 308 patients and 41 controls through a bioinformatics analysis (Table 3 and Data S1).

**TABLE 3:** Genes differentially expressed between T and PT tissues (0.5 ≥ FC ≥ 2; *p* < 0.05).

Genes	T vs PT (18 vs 18)		PT vs CTRL (18 vs 18)		T vs CTRL (18 vs 18)		T vs CTRL (OncoDB) (308 vs 41)	
	*p*‐value*	FC	*p*‐value**	FC	*p*‐value**	FC	*p*‐value***	FC
*IL1B*	0.006	7.79	<0.001	7.18	<0.001	55.98	<0.001	3.60
*CXCL8*	0.012	4.77	<0.001	42.97	<0.001	205.01	<0.001	17.90
*IL1A*	0.014	4.46	0.002	2.63	<0.001	11.72	<0.001	10.06
*CSF2*	0.007	3.66	0.002	2.38	<0.001	8.7	<0.001	8.40
*AGT*	0.002	2.77	<0.001	2.73	<0.001	7.57	<0.001	3.81
*IRAK2*	0.001	2.46	<0.001	11.96	<0.001	29.47	<0.001	3.54
*IRAK1*	<0.001	2.09	<0.001	14.33	<0.001	29.89	<0.001	2.10
*IL1R1*	0.003	0.47	<0.001	7.28	0.001	3.4	0.006	0.75
*TNFSF10*	0.006	0.46	*ns*	–	0.002	0.55	<0.001	0.40
*CHUK*	0.025	0.44	<0.001	7.1	<0.001	3.16	*ns*	–
*TLR6*	0.002	0.41	<0.001	2.68	*ns*	–	*ns*	–
*CSF1*	0.002	0.40	<0.001	5.75	0.002	2.32	<0.001	0.57
*FOS*	0.003	0.37	<0.001	29.36	<0.001	10.75	0.016	0.69
*IL10*	0.004	0.35	<0.001	6.97	0.001	2.43	<0.001	0.58
*TLR3*	<0.001	0.28	*ns*	–	<0.001	0.35	<0.001	0.27
*CCL2*	0.004	0.24	<0.001	8.58	ns	–	*ns*	–
*EGR1*	0.006	0.19	<0.001	127.08	<0.001	24.71	*ns*	–

*Note*: The comparisons between PT vs CTRL and T vs CTRL (our results and OncoDB results) are also reported. The name of the validated genes in OncoDB in the comparison T vs CTRL are underlined. Genes are listed according to descending FC in the T vs PT comparison. *Wilcoxon signed‐rank test; **Mann Whitney test, *** T‐test student.

Abbreviations: CTRL, control; FC, Fold Change; *ns*, not significant; PT, peritumoral; T, tumoral.

**FIGURE 3:**
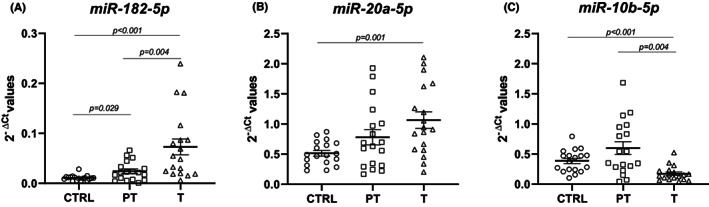
(A) Increased miR‐182‐5p levels in tumoral tissue vs peritumoral and control; (FC = 2.98 and FC = 6.66, respectively). (B) Increased miR‐20a‐5p levels in tumoral tissue vs control; (FC = 2.07). (C) Decreased miR‐10b‐5p levels in tumoral tissue vs peritumoral and control (FC = 0.29 and FC = 0.45, respectively). Data are presented as 2^−∆Ct^ values. Bars represent the expression averages ± standard error of mean (SEM). The *p*‐value was calculated with Wilcoxon signed‐rank test for the comparison T vs PT and with the Mann–Whitney U test for the comparisons T vs CTRL and PT vs CTRL

The qPCR analysis of the selected miRNAs (miR‐20a‐5p, miR‐10b‐5p, and miR‐182‐5p) targeting the four most dysregulated genes (*IL1B*, *CXCL8*, *EGR1*, and *CCL2*) showed that the miR‐182‐5p levels were higher in the T compared with both PT and CTRL, and its levels were higher in PT compared with CTRL. This increasing miRNA expression trend (CTRL‐PT‐T) was observed also for the miR‐20a‐5p, but statistically significant results were found only comparing T with CTRL. The miR‐10b‐5p levels were downregulated in tumoral tissues compared with both PT and CTRL (Table 4 and Figure 3). No difference in miRNAs expression was detected between poorly differentiated (G3) tumours and moderately differentiated (G2) tumours: miR‐182‐5p (*p* = 0.446), miR‐10b‐5p (*p* = 0.599), and miR‐20a‐5p (*p* = 0.599).

**TABLE 4:** miRNAs targeting the most dysregulated genes in T and PT tissues

miRNA	T vs PT		T vs CTRL		PT vs CTRL		Predicted target genes according to DIANA‐TarBase v.8
	*p*‐value*	FC	*p*‐value**	FC	*p*‐value**	FC	
miR‐182‐5p	0.004	2.98	<0.001	6.66	0.029	2.23	*EGR1; CCL2; FOS*
miR‐20a‐5p	*ns*	–	0.001	2.07	*ns*	–	*CXCL8; IRAK2; IL1R1; FOS; CSF1;TNFSF10*
miR‐10b‐5p	0.001	0.29	<0.001	0.45	*ns*	–	*IL1B; IRAK2; FOS; CCL2; EGR1*

*Note*: The comparisons between T vs CTRL and PT vs CTRL are also reported. *Wilcoxon signed‐rank test; **Mann Whitney test.

Abbreviations: CTRL, control; FC, Fold Change; *ns*, not significant; PT, peritumoral; T, tumoral.

## 4 DISCUSSION

In the present study, the expression of 84 genes belonging to the NF‐κB pathway was evaluated in the paired tumoral and peritumoral tissues of patients with CRC compared with normal colonic tissue. The comparison between the tumoral and control tissues revealed, as expected, an upregulation of most of the genes belonging to both the canonical and non‐canonical components of the NF‐κB signalling pathway. In particular, we observed an activation of the IL‐1 signalling pathway with a high upregulation of *IL1A* (FC = 11.72) and *IL1B* (FC = 55.98). *IRAK1* (FC = 29.89) and *IRAK2* (FC = 29.47), two kinases associated with IL1R1, were also upregulated in the tumoral tissue compared to the controls (FC = 3.4), together with *CHUK* (IKKα) (FC = 3.16), whose expression is regulated by IRAK1.

An upregulation of *IL1A*, *IL1B*, *IRAK1*, and *IRAK2* was revealed in tumoral tissues when compared with peritumoral tissues, with lower but significant FC than those identified in the comparison T vs CTRL, suggesting that the PT tissues present an increased grade of inflammation compared to the controls. The upregulation of *IL1B* in T compared with PT was in line with the study conducted by Ping and collaborators, where *IL1B* mRNA levels increased in 33 primary colon cancer samples compared to their matched adjacent normal tissues.^26^ These data were later validated by a bioinformatics analysis with the TCGA database, showing that *IL1B* mRNA levels were significantly increased in CRC patients compared with the controls.^27^ IL‐1 signalling controls CRC development and progression by playing different roles in the tumour microenvironment depending on the cell type. In epithelial cells, IL‐1 pathway drives tumorigenesis without affecting inflammatory responses. The absence of IL1R1 in T cells inhibits the production of IL‐17 and IL‐22, reducing the tumour‐induced inflammation, whereas in myeloid cells it increases the bacterial infiltration into the tumour tissue and triggers a pro‐tumorigenic inflammatory response.^28^

Also, IL‐8 (*CXCL8*), another pro‐inflammatory cytokine, was upregulated in tumoral tissues compared to both peritumoral and control tissues. CXCL8 is a hub gene closely related to CRC carcinogenesis, involved in several steps of the progression and metastasis. By binding its receptors, CXCL8 promotes CRC cell proliferation, invasion, migration, and angiogenesis by activating the PIK3/Akt, MAPK, STAT3, and ERK1/2 signalling cascades.^29^ It induces the epithelial‐mesenchymal transition of cancer cells, contributing to the adhesion and intravasation of CRC cells into the blood. After extravasation, CRC cells induce the mesenchymal‐epithelial transition and form metastases.^30^ The *CXCL8* mRNA levels in tumoral tissues have been correlated with CRC prognosis,^31^, ^32^ poor overall survival, and tumour grade.^33^ In line with our results, a previous study, comparing the gene expression levels of *CXCL8*, identified it as one of the most upregulated genes in tumoral tissues compared to matched normal tissues.^34^

As shown in the heat map (Figure 1), in our case–control study, three TLR members were dysregulated in tumoral tissues compared to control, with an upregulation of the transmembrane receptors *TLR2* and *TLR4* and a downregulation of intracellular *TLR3* that recognizes the viral pathogens in the endosome. TLRs are key molecules involved in inflammation‐driven cancer and all of them, except TLR3, commonly use MYD88 as the downstream adapter protein leading to activation of NF‐κB signalling. Colon carcinogenesis has been associated with increased expression levels of TLR2 and TLR4, which are targets for tumour treatment.^35^ Moreover, multiple single nucleotide polymorphisms within these genes are associated with CRC survival.^36^ Regarding TLR3, negative immunostaining in CRC tissue was recently associated with lymph node metastasis and TLR3 has been indicated as an independent risk factor for recurrence of CRC.^37^

When comparing the gene expression signature of poorly differentiated (G3) tumours with the moderately differentiated (G2) ones, the downregulation of *RELA*, *NOD1*, *CASP8*, *BCL2L1*, *ELK1*, and *IKBKB* was found. These data seem to be in line with those reported by Plewka, et al.^38^ which, by immunohistochemistry, found a higher NF‐κB protein expression in G2 colorectal tumours compared with G1 and G3, suggesting a decreased of NF‐κB in poorly differentiated tumours.

Interestingly, seven genes (*EGR1*, *CCL2*, *IL10*, *FOS*, *CSF1*, *CHUK*, and *IL1R*) were downregulated in tumoral tissues compared to peritumoral tissues, and all of them, except *CCL2*, were upregulated compared to the controls.

Early growth response factor 1 (EGR1) is a transcription factor involved in cell proliferation, differentiation, invasion, and apoptosis and is known to act as tumour‐suppressor or promoter, depending on the cell type and the environment. In CRC cell lines, EGR1 promotes cell growth and inhibits apoptosis.^39^, ^40^, ^41^ These results have also been confirmed in CRC tissues, revealing an association of increased EGR1 expression, both at mRNA and protein levels, with lymphovascular and lymph node invasion, distant metastasis, tumour stage, and poor survival.^42^ In contrast, a recent study showed that increased levels of EGR1inhibited colon cancer cell proliferation, migration, and invasion, whereas knocking down EGR1 increased these parameters.^43^ Therefore, the role of EGR1 in CRC is still unclear and may be context‐specific.^44^

The role of IL10 in CRC is unclear too: some reports indicate that IL10 plays a tumour‐promoting role in CRC,^45^ whereas other studies using a murine tumour model suggest that IL10 suppresses tumour growth.^46^ Indeed, IL10 promotes the secretion of anti‐inflammatory factors in the body but also inhibits effector molecules to achieve tumour immunosuppression.^47^ Although IL10 is a crucial immunosuppression agent, its role in cancer pathogenesis and development is complex and its action as a tumour‐promoting agent or an inhibitor needs to be clarified.^48^

C‐C Motif Chemokine Ligand 2 (CCL2) is a cytokine that has chemotactic activity for monocytes and basophils but not for neutrophils or eosinophils. *CCL2* transcript was found increased in CRC tissues compared with tissues from controls,^49^ and its protein levels were upregulated in adenocarcinoma compared with the control.^50^ Moreover, *CCL2* expression was high in normal colon and CRC fibroblasts and subsets of macrophages in vitro and in vivo paradigms, with its expression being restricted to stromal cells. These data suggest that colon fibroblasts and non‐tumoral cells are the major cellular components that recruit monocytes and establish the macrophage phenotype, characterized by elevated CCL2 production.^51^

Another gene related to macrophages found in our study upregulated in the PT compared with the T tissues is colony‐stimulating factor 1 (*CSF1)*. The CSF1 gene encodes a cytokine that controls the production, differentiation, and function of macrophages. Previous studies found overexpression of the CSF1 protein exclusively in colon cancer cells, which correlated with macrophage infiltration.^52^ Macrophage infiltration has been suggested as an independent poor prognostic factor in several types of cancers. Blockade of CSF1 or its receptor proved to be a selective approach to manipulating tumour‐associated macrophages in different types of cancer.^53^

Fos Proto‐Oncogene, AP‐1 Transcription Factor Subunit (FOS) encodes leucine zipper proteins that dimerizing with other proteins belonging to the JUN family, form the transcription factor complex AP‐1. It is a proto‐oncogene primarily involved in cell differentiation, signal transduction, and proliferation.^54^ Regarding CRC, c‐Fos is involved in IL6 and VEGF‐A transcription.^55^ Moreover, c‐Myb promotes growth and metastasis of CRC through c‐fos‐induced epithelial‐mesenchymal transition.^56^

Other two genes found downregulated in tumoral tissue compared with peritumoral, but upregulated compared with the controls were *IL1R1* and *CHUK* both belonging to the IL‐1 signalling pathway and whose involvement in CRC has been discussed above.

The higher transcription levels of NF‐κB‐related genes in peritumoral compared with tumoral tissues indicate that inflammation and immune response are also activated in the non‐affected adjacent tissue. The peritumoral inflammatory reaction in colon cancer has been found by histological and immunohistochemical methods, showing that the inflammatory infiltrate is mainly formed by lymphocytes, plasma cells, and macrophages.^57^

In the last part of the study, we evaluated the expression of miRNAs targeting the most dysregulated genes in T, PT, and CTRL. We found that miR‐20a‐5p and miR‐182‐5p were upregulated in T compared with PT tissues, whereas miR‐10b‐5p was downregulated in T compared to PT and control tissues. The upregulation of miR‐20a‐5p is in agreement with the literature, where higher miR‐20a‐5p levels were found in cancer tissue compared with peritumoral tissue, and it was also significantly overexpressed in faecal samples of CRC patients compared with healthy controls.^58^ Its levels correlated with survival^59^ and predicted a poor prognosis in CRC evolution.^60^ Moreover, miR‐20a‐5p has been associated with tumour localization, stroma abundance, tumour grade, and peritumoral inflammatory infiltrate, while tumour stage and progression were independent progression factors in CRC.^61^

Our study also revealed increased levels of miR‐182‐5p in tumoral tissues compared with both peritumoral samples and normal colonic mucosa. This was in contrast to previous studies, where miR‐182‐5p expression was significantly decreased in CRC tissue samples and cell lines, and its upregulation led to inhibition of cell proliferation, invasion, and migration ability of CRC cells.^62^ Moreover, miR‐182‐5p was found to reduce the proportion of apoptotic cells.^63^ However, this miRNA was significantly overexpressed in the plasma of CRC patients compared with the controls.^64^

Regarding the expression of miR‐10b‐5p in CRC, its levels were downregulated in tumoral tissues compared with peritumoral and control samples. High expression of miR‐10b‐5p was associated with tumours located in the right colon relative to the left colon and rectum.^65^ In addition, a study in a cell line showed that an inhibition of CRC growth was induced by miR‐10b‐5p knockdown and matrine treatment, suggesting that this natural product could suppress proliferation, migration, and invasion of CRC cells through the miR‐10b/PTEN pathway.^66^

It is well known that miRNAs mainly downregulate gene expression by targeting mRNAs. In this study, miR‐182‐5p was upregulated, while *EGR1* and *CCL2* were downregulated in T compared with PT tissues. On the contrary, miR10b‐5p was downregulated, while *IL1B* was overexpressed in T compared with both PT and CTRL. However, our results cannot prove that miR‐182‐5p and miR‐10b‐5p regulate the expression of the predictive targets *EGR1*, *IL1B*, and *CCL2*, although they showed an opposite trend of expression. Further studies on large cohorts are needed to confirm these results.

## 5 CONCLUSIONS

Our results may contribute to the design of new experimental strategies based on endogenous molecules, such as miRNAs, to target the key players of the NF‐ κB pathway. The identification of novel biomarkers of early diagnosis and response to specific treatments together with new approaches for CRC therapy are still open challenges.

## AUTHOR CONTRIBUTIONS

**Maria Dobre:** Conceptualization (equal); funding acquisition (lead); investigation (equal); writing – review and editing (equal). **Bogdan Trandafir:** Resources (equal); writing – original draft (equal). **Elena Milanesi:** Data curation (lead); methodology (lead); visualization (lead); writing – original draft (equal). **Alessandro Salvi:** Writing – review and editing (equal). **Ioana Alina Bucuroiu:** Writing – review and editing (equal). **Catalin Vasilescu:** Resources (equal); writing – review and editing (equal). **Andrei Marian Niculae:** Investigation (equal); writing – review and editing (equal). **Vlad Herlea:** Investigation (equal); writing – review and editing (equal). **Mihail Eugen Hinescu:** Conceptualization (equal); writing – review and editing (equal). **Gabriel Constantinescu:** Supervision (lead); writing – review and editing (equal).

## CONFLICT OF INTEREST

The authors declare that they have no competing interests.

## Supporting information


Table S1

Data S1

## Data Availability

Raw data can be obtained from the corresponding author upon reasonable request.
