# Supplementary material for: Molecular profile of the NF‐κB signalling pathway in human colorectal cancer
Source: J Cell Mol Med. 2022 Nov 25;26(24):5966–75. doi: 10.1111/jcmm.17545 (PMC9753446; doi:10.1111/jcmm.17545)
Supplement: Supplementary file 1 — Table S1 [file JCMM-26-5966-s002.docx]

**Supplementary Table 1** Genes involved in NFkB Signaling Pathway (PAHS-025Z, Qiagen)

| ***Ligands and receptors:*** *CD40 (TNFRSF5), CD83, EGFR, FASLG (TNFSF6), IL10, IL1A, IL1B, IL1R1, IL8, LTBR, NOD1 (CARD4), TLR1, TLR2, TLR3, TLR4, TLR6, TLR9, TNF, TNFRSF1A, TNFRSF10A, TNFRSF10B, TNFSF10.* |
| --- |
| ***Cytoplasmic sequestering/releasing of NF-κB:*** *BCL3, CHUK (IKKa), IKBKB, IKBKG, NFKBIA, NFKBIE.* |
| ***Transcription factors:*** *NFKB1, NFKB2, REL, RELA, RELB.* |
| ***Downstream signalling:*** *BIRC2 (c-IAP2), FADD, IRAK1, IRAK2, IRF1, MYD88, RIPK1, TBK1, TICAM1 (TRIF), TNFAIP3, TRADD, TRAF2, TRAF3, TRAF6.* |
| ***General immune response:*** *CCL2 (MCP-1), CCL5 (RANTES), CSF1 (MCSF), CSF2 (GM-CSF), CSF3 (GCSF), ICAM1, IFNA1, IFNG, IL8, LTA (TNFB), TNF.* |
| ***Apoptosis:*** *AGT, BCL2A1 (BCL-X), BCL2L1, BIRC3 (c-IAP1).* |
| ***Other factors involved in the NF-κB pathway:***  Kinases: *AKT1, MAP3K1, RAF1.*  Transcription factors: *ATF1, EGR1, ELK1, FOS, JUN, STAT1.*  Other genes: *CARD11, CASP1 (ICE), CASP8, CFLAR (CASPER), HMOX1, MALT1, PSIP1, RHOA, TIMP1.*  Pathway Activity Signature Genes:  *BIRC3, CCL20, CD83, CXCL2, CXCL3, ICAM1, IL8, IRF1, NF-KB1, NF-KBIA, NF-KBIE, STX11, TIFA, TNF, TNFAIP2, TNFAIP3*. |
| ***Housekeeping genes :*** *ACTB, HPRT1, GAPDH, B2M, RPLP0,* |
